# Supplementary figures and images for: Control of protein synthesis and memory by GluN3A-NMDA receptors through inhibition of GIT1/mTORC1 assembly
Source: eLife. 2021 Nov 17;10:e71575. doi: 10.7554/eLife.71575 (PMC8598234; doi:10.7554/eLife.71575)

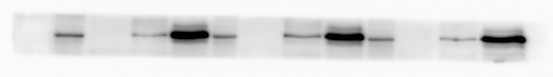

Supplement: Figure 6—figure supplement 1—source data 1. [file elife-71575-fig6-figsupp1-data1.zip › Figure 6 - figure supplement 1/Orig-Scan_Figure6-Figuresuppl1_source-data_1/GIT1.png]

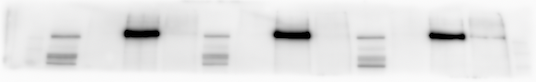

Supplement: Figure 6—figure supplement 1—source data 1. [file elife-71575-fig6-figsupp1-data1.zip › Figure 6 - figure supplement 1/Orig-Scan_Figure6-Figuresuppl1_source-data_1/mTOR.png]

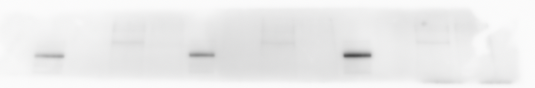

Supplement: Figure 6—figure supplement 1—source data 1. [file elife-71575-fig6-figsupp1-data1.zip › Figure 6 - figure supplement 1/Orig-Scan_Figure6-Figuresuppl1_source-data_1/GluN2AB.png]

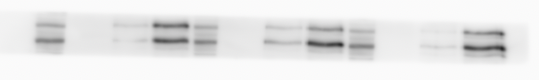

Supplement: Figure 6—figure supplement 1—source data 1. [file elife-71575-fig6-figsupp1-data1.zip › Figure 6 - figure supplement 1/Orig-Scan_Figure6-Figuresuppl1_source-data_1/bPIX.png]

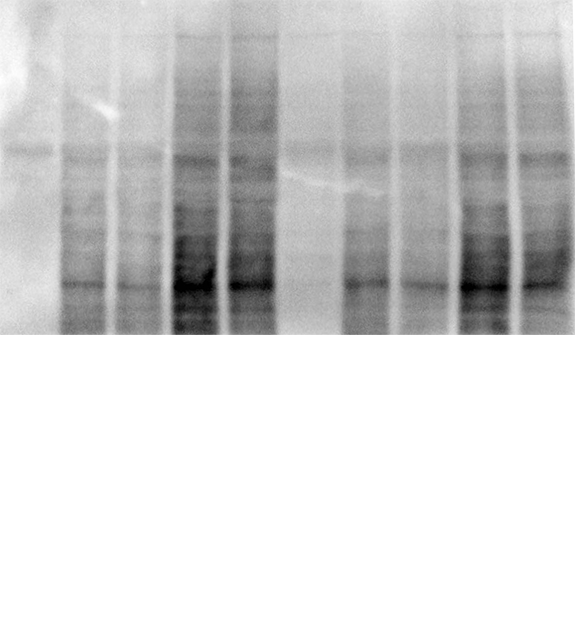

Supplement: Figure 6—figure supplement 2—source data 1. [file elife-71575-fig6-figsupp2-data1.zip › Figure 6 - figure supplement 2/Orig-Scan_Figure6-Figuresuppl2_source-data_1/#SUnSET.tif]
